# Genomic characterization of an emerging *bla*_KPC-2_ carrying Enterobacteriaceae clinical isolates in Thailand

**DOI:** 10.1038/s41598-019-55008-x

**Published:** 2019-12-06

**Authors:** Anusak Kerdsin, Saowarat Deekae, Sunee Chayangsu, Rujirat Hatrongjit, Peechanika Chopjitt, Dan Takeuchi, Yukihiro Akeda, Kazunori Tomono, Shigeyuki Hamada

**Affiliations:** 1 Faculty of Public Health, Kasetsart University, Chalermphrakiat Sakon Nakhon Province Campus, Sakon Nakhon, Thailand; 2 Surin Hospital, Surin, Thailand; 3 Faculty of Science and Engineering, Kasetsart University, Chalermphrakiat Sakon Nakhon Province Campus, Sakon Nakhon, Thailand; 4 Japan-Thailand Research Collaboration Center for Infectious Diseases, Research Institute for Microbial Diseases, Osaka University, Osaka, Japan; 5 Department of Infection Control and Prevention, Graduate School of Medicine, Osaka University, Osaka, Japan; 6 Division of Infection Control and Prevention, Osaka University Hospital, Osaka University, Osaka, Japan

**Keywords:** Microbiology, Clinical microbiology

## Abstract

The rapidly increasing prevalence of carbapenem-resistant Enterobacteriaceae (CRE) over the past decade has increased concern in healthcare facilities and the impact on public health. The prevalence of *bla*_KPC_ (KPC) in Thailand remains very low; only *bla*_KPC-13_ has been described previously. This study is the first to describe the characteristics of *bla*_KPC-2_-carrying *Klebsiella pneumoniae*, *Escherichia coli*, and *Enterobacter asburiae* in Thailand. The prevalence rate of *bla*_KPC-2_-carrying isolates was 0.13% among CRE isolates in our study. Based on carbapenem susceptibility testing, *K. pneumoniae* C1985 was resistant to meropenem and ertapenem, *E. coli* C1992 was resistant to meropenem, imipenem, and ertapenem, and *E. asburiae* C2135 was only resistant to imipenem. *K. pneumoniae* C1985 carried *bla*_*KPC-2*_*, bla*_*SHV-11*_*, fosA, oqxA*, and *oqxB*, while *E. coli* C1992 contained *bla*_*KPC-2*_ and *mdf(A)* and *E. asburiae* C2135 harbored *bla*_*KPC-2*_*, bla*_*ACT-2*_, and *qnrE1*. The genetic features of *bla*_KPC-2_ in the 3 isolates revealed identical rearrangement and flanking regions. Analysis of genomic sequences from these 3 isolates revealed that the sequence types of *K. pneumoniae* C1985, *E. coli* C1992, and *E. asburiae* C2135 were ST4008, ST7297, and ST1249, respectively. The 3 *bla*_*KPC-2*_ isolates were from individual living cases. Two cases were colonization for *K. pneumoniae* C1985 and *E. asburiae* C2135 and the third case was hospital-acquired infection of *E. coli* C1992. Although the prevalence of *bla*_KPC-2_-carrying CRE is relatively low in this study, continued surveillance and close monitoring are warranted. In addition, prompt or early detection of CRE and strict implementation of infection control are essential to prevent outbreaks or rapid spread in hospitals.

## Introduction

The rapidly increasing prevalence of carbapenem-resistant Enterobacteriaceae (CRE) over the past decade has increased concern in healthcare facilities and public health communities worldwide^1,2^. CRE such as *Escherichia coli*, *Klebsiella pneumoniae*, *Enterobacter* spp., and *Citrobacter* spp. produce a variety of carbapenemases. There are three main groups of enzymes responsible for most carbapenem resistance: KPC (*Klebsiella pneumoniae* carbapenemase) (Ambler class A), Metallo-ß-Lactamases (Ambler class B) such as NDM and IMP, and OXA-48-like (Ambler class D)^2^. These enzymes are encoded by *bla*_KPC_, *bla*_NDM_, *bla*_IMP_, and *bla*_OXA48-like_, respectively, and are frequently found worldwide^2^. In Thailand, the prevalence of CRE during 2000–2018 increased from 1% to 10.2% for imipenem and from 1.2% to 10.2% for meropenem in *K. pneumoniae*, while the resistance rate in *E. coli* increased from 1% to 2.4% for imipenem and from 0.6% to 2.6% for meropenem, respectively (http://narst.dmsc.moph.go.th/). Carbapenemase genes have been detected in Enterobacteriaceae in Thailand including *bla*_NDM-1_, *bla*_IMP-14a_, *bla*_KPC-13_, *bla*_OXA-48_, *bla*_OXA-181_, and *bla*_OXA-232_^3–6^.

KPC was first reported in *K. pneumoniae* from the USA in 1996 and is endemic in the USA, Colombia, Argentina, Israel, Greece, Italy, and China^2^. In Thailand, KPC-13 (*bla*_KPC-13_) was first described in Bangkok in 2014; however, other KPC types have not been detected^4^. The current study is the first to describe the characteristics of KPC-2-carrying *K. pneumoniae, E. coli*, and *Enterobacter asburiae* from rural areas in Thailand.

## Results

### Clinical information of *bla*_KPC-2_-carrying patients

The 3 *bla*_*KPC-2*_ isolates were from individual living cases. *K. pneumoniae* C1985, *E. coli* C1992, and *E. asburiae* C2135 were isolated from urine of a 67-year-old female, from pus in the right arm of a 45-year-old male, and from sputum of a 43-year-old female, respectively. The diagnosis of the 45-year-old male was cellulitis in the right arm with septicemia; this case was classified as a hospital-acquired infection of *E. coli* C1992. Hospitalization of this case was at internal medicine for 14 days in September 2018. The diagnosis of the 67-year-old female was cholangiocarcinoma and this case was classified as colonization by *K. pneumoniae* C1985. This patient was admitted to the surgery ward during December 2018 (5 days). Finally, a breast cancer was diagnosed in the 43-year-old female and this case was classified as colonization by *E. asburiae* C2135 and the patient was admitted to the cancer ward for 14 days between March and April 2019. Meropenem treatment was used for the 45-year-old male (*E. coli* C1992), while the 67-year-old female (*K. pneumoniae* C1985) was treated with piperacillin-tazobactam and then this was changed to meropenem. No antibiotics were used to treat the *E. asburiae* C2135 case in the 43-year-old female.

### Genomic characterization of *bla*_*KPC-2*_*-*carrying isolates

In September 2015, we established a surveillance network to detect carbapenemase genes in CRE isolates from hospitals in 11 provinces in Thailand consisting of: Surin, Udonthani, Sakhon Nakhon, Nakhon Phanom, Roi Et, Bueng Kan, Mukdahan, Tak, Phayao, Chumporn, and Surat Thani. The network is still operational. Of 2,245 CRE isolates, 3 isolates harboring *bla*_*KPC*_ (0.13%) were detected using multiplex PCR. These isolates were from a hospital in Surin province, northeastern Thailand. Biochemical identification revealed 2 isolates were *K. pneumoniae* (strains C1985 and C2135) and the other isolate was *E. coli* (strain C1992). However, matrix-assisted laser desorption ionization-time of flight mass spectrometry (MALDI-TOF MS Biotyper, Bruker, Daltonics, Germany) identification revealed isolates no. C1985, C1992, and C2135 were *K. pneumoniae*, *E. coli*, and *E. asburiae*, respectively. Comparison of genomic sequences using KmerFinder and the ANI calculator demonstrated that isolate C2135 was *Enterobacter asburiae*, while isolate C1985 was *K. pneumoniae* and isolate C1992 was *E. coli*.

**Figure 1:**
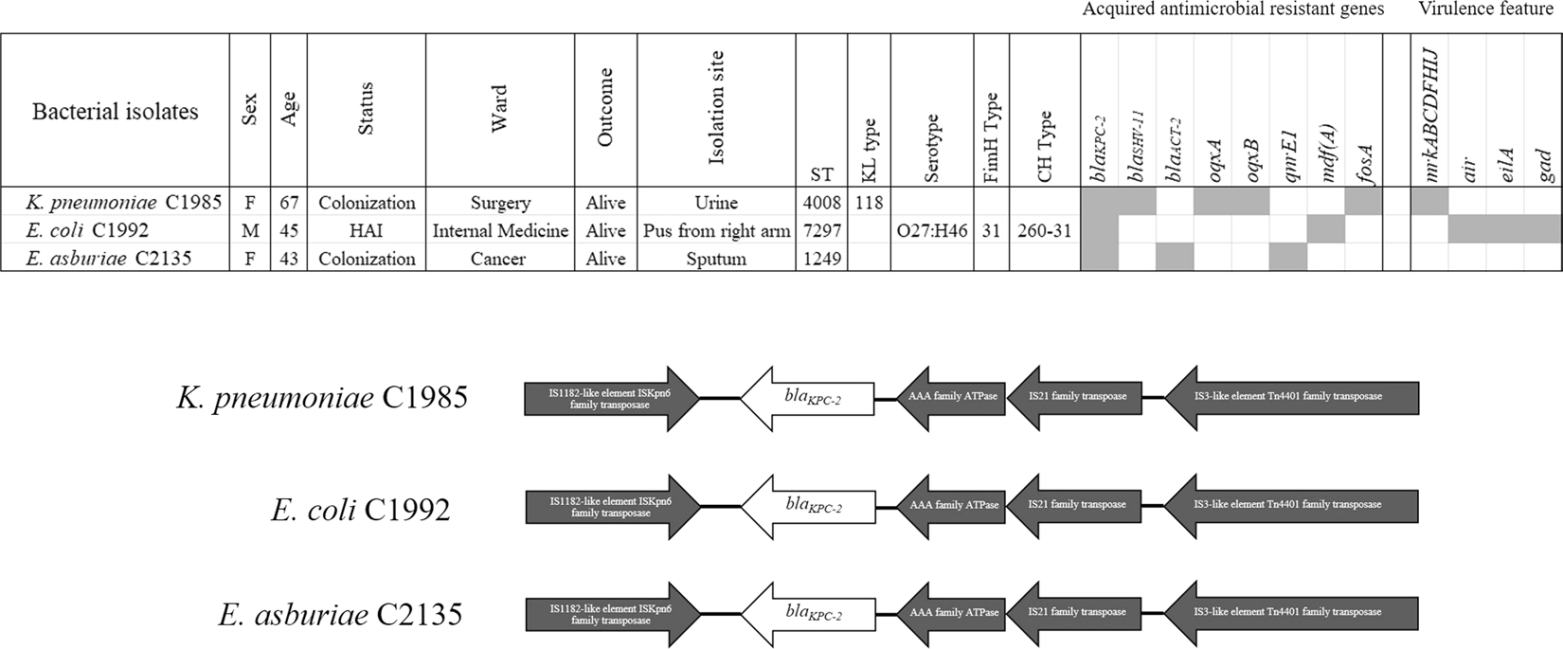
Characteristics of *bla*_KPC-2_-carrying Enterobacteriaceae isolates and genetic organization of *bla*_KPC-2_ in *K. pneumoniae* C1985, *E. coli* C1992, and *E. asburiae* C2135.

The MIC values for the 18 antimicrobial agents are shown in Table 1, *K. pneumoniae* C1985 was resistant to 4 agents (amoxicillin/clavulanic acid, cefotaxime, ceftriaxone, and piperacillin/tazobactam). This isolate was intermediately resistant to meropenem and ertapenem. *E. coli* C1992 was resistant to 8 antibiotics (amoxicillin/clavulanic acid, cefotaxime, ceftazidime, ceftriaxone, meropenem, imipenem, ertapenem, and piperacillin/tazobactam). Finally, *E. asburiae* C2135 was resistant to amoxicillin/clavulanic acid, cefotaxime, ceftriaxone, imipenem, and piperacillin/tazobactam. ResFinder and CARD analysis of all genomic isolates showed 5 acquired antimicrobial-resistant genes in *K. pneumoniae* C1985, 2 acquired antimicrobial-resistant genes in *E. coli* C1992, and 3 acquired antimicrobial-resistant genes in *E. asburiae* C2135, respectively (Fig. 1). *K. pneumoniae* C1985 carried *bla*_*KPC-2*_*, bla*_*SHV-11*_*, fosA, oqxA*, and *oqxB*, while *E. coli* C1992 contained *bla*_*KPC-2*_ and *mdf(A)*. *E. asburiae* C2135 harbored *bla*_*KPC-2*_*, bla*_*ACT-2*_, and *qnrE1*. The genetic features of *bla*_KPC-2_ in 3 isolates revealed identical rearrangement and flanking regions (Fig. 1). Analysis of serotype, FimH type, and CH type in *E. coli* C1992 revealed O27:H46, *fimH31*, and 260–31, respectively. The KL type (polysaccharide capsule and lipopolysaccharide O antigen) of *K. pneumoniae* C1985 showed KL118 using Kaptive software analysis. Virulence-associated genes analysis showed that *K. pneumoniae* C1985 contained only *mrkABCDFHIJ*, while *E. coli* C1992 carried *air*, *eilA*, and *gad* (Fig. 1).

**Table 1:** Susceptibility profiles of all *bla*_KPC-2_-containing isolates.

Antimicrobials	*K. pneumoniae* C1985		*E. coli* C1992		*E. asburiae* C2135	
	MIC	Interpreted	MIC	Interpreted	MIC	Interpreted
Amoxicillin/clavulanic acid	>16	R	>16	R	>16	R
Piperacillin/tazobactam	>64	R	>64	R	>64	R
Cefepime	2	S	8	SDD	2	S
Cefotaxime	4	R	16	R	8	R
Ceftazidime	8	I	16	R	4	S
Ceftriaxone	16	R	>32	R	32	R
Ertapenem	1	I	4	R	≤0.5	S
Imipenem	1	S	4	R	4	R
Meropenem	2	I	4	R	2	I
Doripenem	1	S	2	I	≤0.5	S
Amikacin	≤8	S	≤8	S	≤8	S
Gentamicin	≤2	S	≤2	S	≤2	S
Ciprofloxacin	≤0.06	S	≤0.06	S	≤0.06	S
Levofloxacin	≤0.06	S	≤0.06	S	≤0.06	S
Netilmicin	≤8	S	≤8	S	≤8	S
Trimethoprim/sulfamethoxazole	≤1	S	≤1	S	≤1	S
Colistin	≤1	WT or S*	≤1	WT or S*	≤1	WT or S*
Tigecycline	≤0.25	S*	≤0.25	S*	0.5	S*

R = resistance; I = intermediate; S = susceptible; SDD = susceptible dependent dose; WT = wild type.

*interpret following EUCAST, 2019.

**Figure 2:**
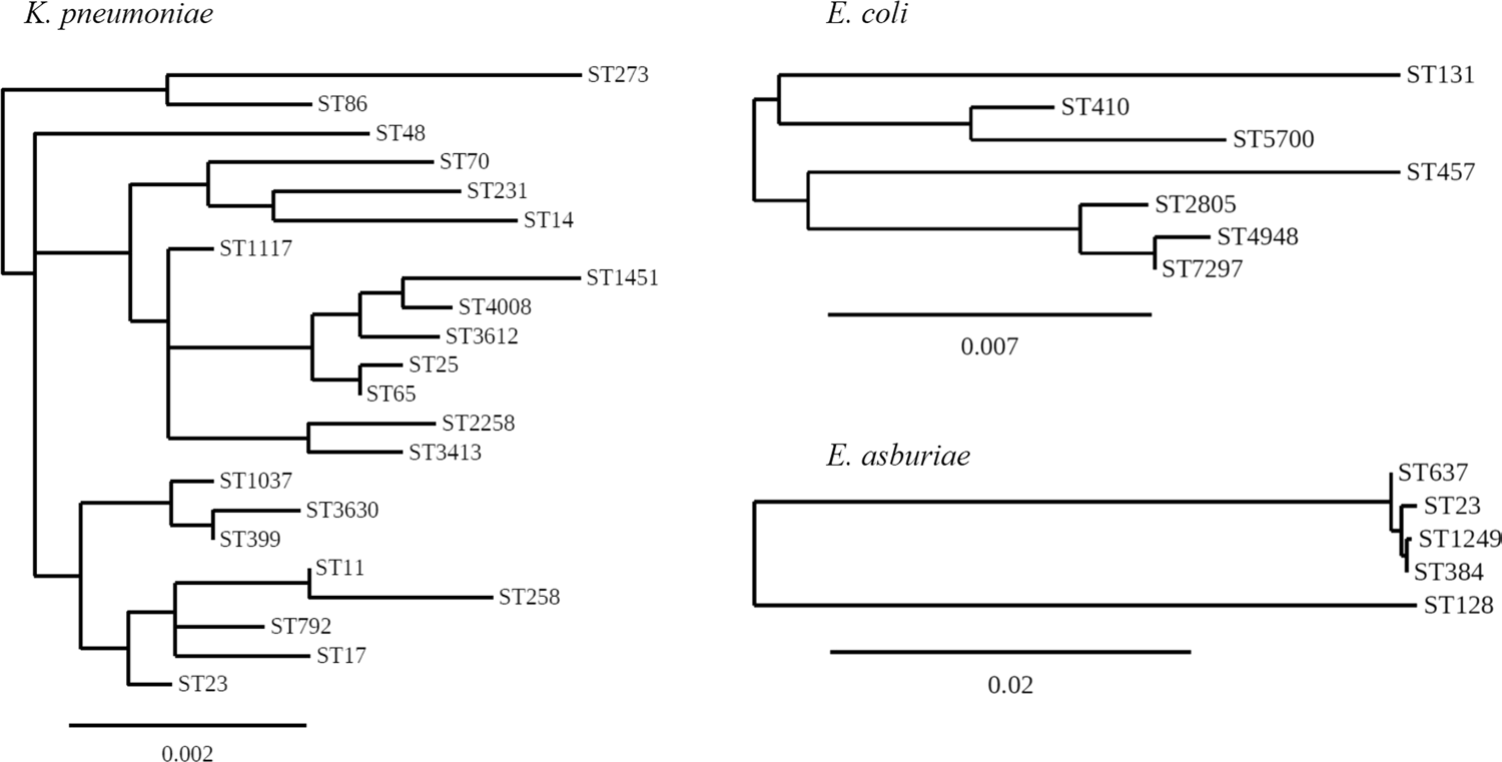
Unrooted tree based on the alignments of concatenated MLST allelic sequences in 3 species *K. pneumoniae* ST4008 (C1985)*, E. coli* ST7297 (C1992), and *E. asburiae* ST1249 (C2135) using the neighbor-joining method. Scale bar indicates sequence dissimilarity.

Analysis of genomic sequences from these 3 isolates revealed the sequence types (STs) of *K. pneumoniae* C1985, *E. coli* C1992, and *E. asburiae* C2135 were ST4008, ST7297, and ST1249, respectively. *K. pneumoniae* ST4008 and *E. asburiae* ST1249 are novel STs. Analysis based on the MLST database revealed that *K. pneumoniae* (C1985) ST4008 was a double-locus variant of *gapA* and *tonB* for ST399, ST3630 and ST3413, *mdh* and *tonB* for ST1037, *phoE* and *rpoB* for ST1451, *phoE* and *tonB* for ST2258, and *gapA* and *rpoB* for ST3612. The phylogenetic tree constructed using concatenated sequences of the 7 MLST genes showed that ST4008 was very close to ST1451 (Fig. 2). *E. coli* (C1992) ST7297 was closely related to ST4948 by a single locus variant (*mdh*) and this ST was a double locus variant (*gyrB* and *icd*) of ST2805 concordant with phylogeny analysis (Fig. 2). *E. asburiae* (C2135) ST1249 was closely related to *E. cloacae* ST384 with a single locus variant (*dnaA*) and this ST had no double-locus variant with any of the STs in the MLST database (Fig. 2). A triple locus variant of ST1249 was found in ST23 and ST637. The phylogenetic tree indicated that ST1249 was closely related to ST384 (Fig. 2).

**Figure 3:**
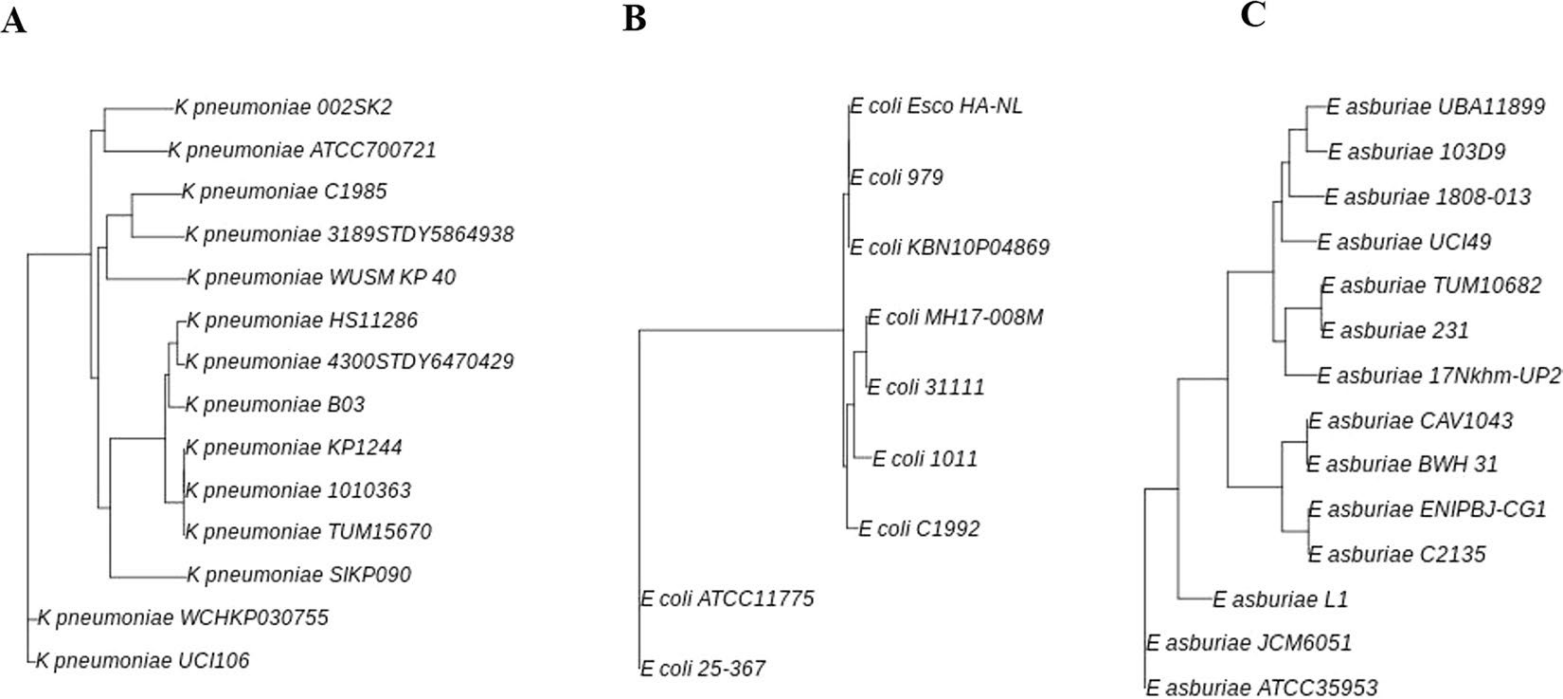
Unrooted tree based on single nucleotide polymorphism (SNP) in 3 species *K. pneumoniae* ST4008 (C1985)*, E. coli* ST7297 (C1992), and *E. asburiae* ST1249 (C2135) using the neighbor-joining method.

As shown in Fig. 3, based on a reference genome-based single nucleotide polymorphism (SNP) *K. pneumoniae* C1985 was related to *K. pneumoniae* strain 3189STDY5864938 (ST2258) isolated from human blood in Pakistan. *E. coli* C1992 was more related to *E. coli* strain 1011 than strains 31111 and MH17–008M. The strain 1011 was isolated from urine of patient with urinary infection in Germany, whereas strains 31111 and MH17–008M were from Vietnam, having been isolated from human feces and blood, respectively. *E. asburiae* C2135 isolate was related to *E. asburiae* strain ENIPBJ-CG1; this isolate was from a bone marrow transplant patient in China.

## Discussion

Due to a rise in CRE globally, there are gradually fewer options for treatment. Carbapenemases is a major key mechanism for resistance to carbapenems and other *β*-lactam antimicrobial agents. The prevalence of CRE and the carbapenemase species such as KPC, NDM, IMP, and OXA-48 family enzymes is dependent on geographic region. KPC and its variants are mostly found in the USA, South America, and European countries^7^. NDM is concentrated in Asia (India, Pakistan, Bangladesh, China), and Southeast Asian countries^7^. The OXA-48-like enzymes are mainly found in European countries (France, Germany, the Netherlands, Italy, and the United Kingdom), in the Middle East (Turkey), and in Mediterranean countries including North Africa (mainly Morocco, Tunisia, Egypt, and Libya)^7^. In Thailand, *bla*_NDM_, *bla*_OXA48-like_ and *bla*_IMP-14_ are frequently detected in clinical Enterobacteriaceae isolates^3,6^.

However, the prevalence of *bla*_KPC_ (KPC) in Thailand remains very low; only *bla*_KPC-13_ has been described previously^4,8^, with the later study reporting rates of *bla*_KPC-13_-carrying isolates of 0.02% among Enterobacteriaceae and 1.7% among CRE isolates. This current study is the second report on KPC in Thailand and we discovered *bla*_KPC-2_ for the first time in Thai patients. The prevalence rate of *bla*_KPC-2_-carrying isolates was 0.13% among CRE isolates (n = 2,245) in our study, which was lower than in the previous report^8^. Other reports showed high prevalence rates of *bla*_KPC-2_-carrying Enterobacteriaceae of 14.6% in Taiwan^9^, 71.4% in Eastern China^10^, 25.3% in Australia^11^, and about 40–65% in Greece^12^. The *bla*_KPC-2_-carrying patients in the current study were separately admitted in different wards, indicating that there was no relationship or contact among them. Indeed, the genetic organization of *bla*_KPC-2_ was similar among the isolates even though they were different species. We hypothesized that these isolates may have received this gene from the same source. However, complete plasmid sequences should be done and aligned to confirm this hypothesis. In addition, further study should investigate for ability of *bla*_KPC-2_ conjugative transferable and enzyme kinetics.

The *bla*_KPC_-carrying *K. pneumoniae* strains can be characterized by their clonality, in that the majority of the strains circulating globally belong to clonal complex (CC) 258 as defined by MLST. The most common ST is ST258 in the USA, but other STs in CC258 such as ST11, ST340, ST437, and ST512 predominate in countries outside the USA^1^. In Singapore, *bla*_KPC-2_-carrying *K. pneumoniae* were associated with non-ST258 isolates, namely ST11, ST14, ST17, ST23, ST48, ST65, ST70, ST86, ST231, ST273, ST792, and ST1117^13^. Vargas *et al*. (2019) reported 40% of *K. pneumoniae* ST25 (CC65) carried *bla*_KPC-2_ in Argentina. In China, *bla*_KPC-2_-carrying ST11 in *K. pneumoniae* isolates were the most frequent^10,14^. *K. pneumoniae* ST86 was reported to be carrying *bla*_KPC-2_ in Canada^15^. Our study was different from those reports, because we revealed a novel ST4008 of *K. pneumoniae* harboring *bla*_KPC-2_. This ST was closely related to ST1451, a strain susceptible to carbapenem and isolated from human blood in North America (https://bigsdb.pasteur.fr/cgi-bin/bigsdb/bigsdb.pl?db=pubmlst_klebsiella_seqdef&page=query). SNP phylogeny demonstrated that *K. pneumoniae* C1985 (ST4008) is related to strain 3189STDY5864938 from Pakistan; this is ST2258. MLST revealed that our isolate was closely related to ST1451; however, the genomic sequence of ST1451 is not available in the database yet, so comparison to these genomes is not possible at this time.

*E. coli* carrying *bla*_KPC-2_ has been reported in ST131, ST410, ST457, and ST5700 from Italy, Greece, China, Mexico, and Tunisia^16–21^, respectively, whereas our study involved detection in ST7297. This ST is closely related to ST4948 and both have the same ancestor similar with ST2805 according to the phylogenetic tree of concatenated MLST allelic sequences. SNP analysis revealed that *E. coli* C1992 (ST7297) was related to *E. coli* strain 1011 isolated from Germany; this strain is ST131. Due to there being no genomic sequence of ST4948 in the database yet, comparison between the genomes of these isolates is not possible at this time. Finally, *bla*_KPC-2_ was found in several species of Enterobacter such as *E. asburiae, E. cloacae*, and *E. hormaechei*^22–24^, similar to our study. SNP phylogeny revealed that *E. asburiae* in our study was related to the isolate from China.

In conclusion, we reported the emergence of *bla*_KPC-2_-harboring Enterobacteriaceae isolates. Although the prevalence of this type of CRE is relatively low, continued surveillance and close monitoring are warranted. In addition, prompt or early detection of CRE and strict implementation of infection control are essential to prevent outbreaks or rapid spread in hospitals.

## Methods

### Bacterial isolates and case information

Three isolates (*K. pneumoniae* C1985, *E. coli* C1992, and *E. asburiae* C2135) each carrying *bla*_KPC-2_ were used to characterize this study. Clinical information of patients with these isolates was reviewed by the attending physicians at the hospital.

### Microbiological methods

Presumptive conventional biochemical tests described elsewhere were used for presumptive *K. pneumoniae* and *Enterobacter* spp., identification, including indole production, methyl-red reaction, Voges-Proskauer reaction, citrate utilization, and urea hydrolysis^25^. An *E. coli* identification kit (U & V Holding, Thailand) was used for confirmation of *E. coli* according to the manufacturer’s instruction. MALDI-TOF MS Biotyper (Bruker, Daltonics, Germany) was used to confirm *E. coli*, *K. pneumoniae*, and *E. asburiae*. Carbapenemase genes were detected using multiplex PCR^26^.

The minimal inhibition concentrations (MICs) for 18 antimicrobial agents (amoxicillin/clavulanic acid, amikacin, cefepime, cefotaxime, ceftazidime, ceftriaxone, ciprofloxacin, doripenem, ertapenem, meropenem, imipenem, gentamicin, levofloxacin, netilmicin, piperacillin/tazobactam, trimethroprim/sulfamethoxazole, tigecycline, and colistin) were determined among 3 isolates using broth microdilution and the antimicrobial susceptibility profile (resistant, intermediate, and susceptible) with interpretation according to the Clinical Laboratory Standards Institute^27^, except for tigecycline which was interpreted using European Committee on Antimicrobial Susceptibility Testing (EUCAST)^28^.

### Whole genome sequencing and analysis

The whole genome sequencing of the three *bla*_KPC-2_ isolates was carried out on the Illumina platform with massively parallel sequencing Illumina technology at Beijing Novogene Bioinformatics Technology Co., Ltd. (Beijing, China). A-tailed, ligated to paired-end adaptors and PCR amplified with a 500 bp insert and a mate-pair library with an insert size of 5 kb were used for the library construction.

Illumina PCR adapter reads and low-quality reads from the paired-end and mate pair library were filtered by the step of quality control using the company compiling pipeline. All good quality paired reads were assembled using the SOAP de novo (http://soap.genomics.org.cn/soapdenovo.html) into a number of scaffolds. The assembly result was gap-filled and optimized by the company using software such as krskgf and gapclose.

Analysis of whole genome sequence data was performed for confirmation of the species using KmerFinder 3.1 (https://cge.cbs.dtu.dk/services/KmerFinder/) and the ANI calculator (https://www.ezbiocloud.net/tools/ani)^29,30^. Antimicrobial resistance genes were identified using ResFinder 3.1 (https://cge.cbs.dtu.dk/services/ResFinder/) and The Comprehensive Antibiotic Resistance Database (CARD) (https://card.mcmaster.ca/)^31,32^. Serotypes, CH type, FimH type, KL type, and virulence genes were analyzed using SerotypeFinder 2.0 (https://cge.cbs.dtu.dk/services/SerotypeFinder/), CH typer (https://cge.cbs.dtu.dk/services/CHTyper/), FimTyper 1.0 (https://cge.cbs.dtu.dk/services/FimTyper/), Kaptive (http://kaptive.holtlab.net/), and VirulenceFinder 2.0 (https://cge.cbs.dtu.dk/services/VirulenceFinder/), Institut Pasteur (https://bigsdb.pasteur.fr/klebsiella/klebsiella.html), respectively^33–37^.

Multilocus sequence typing (MLST) analysis for sequence types of *E. coli, E. cloacae* complex, and *K. pneumoniae* used MLST 2.0 (https://cge.cbs.dtu.dk/services/MLST/)^38^ and Institut Pasteur MLST (https://bigsdb.pasteur.fr/klebsiella/klebsiella.html), respectively. Construction of phylogenetic trees for all STs in this study was performed via Phylogeny.fr^39^. The genomic comparison of these isolates was conducted using a reference genome-based single nucleotide polymorphism (SNP) strategy with BacWGSTdb^40^ (http://bacdb.org/BacWGSTdb/).

### Accession number

The genomic sequences assembled were deposited under the Bioproject accession number of PRJNA525849 with Sequence Read Archive (SRA) accession numbers of SRR8690013 (plasmid contigs) and SRR9722215 (chromosomal contigs) for *K. pneumoniae* C1985. The genomic sequence of *E. coli* C1992 was deposited under the Bioproject accession number of PRJNA525942 with SRA accession numbers of SRR8693447 (plasmid contigs) and SRR9722217 (chromosomal contigs), respectively. The genomic sequence of *E. asburiae* C2135 was deposited under the Bioproject accession number of PRJNA555780 with the SRA accession number of SRR9729532.

### Ethical approval

This study was reviewed and approved by the Surin Hospital Ethics Review Board (ERB). The medical records of these 3 cases were reviewed by attending physicians at the hospital using the clinical case record form approved by the ERB. The ERB waived requirement for informed consent as the study satisfied the conditions of the policy statement on ethical conduct for research involving humans. This study was conducted according to the principles expressed in the Declaration of Helsinki.
